# An Overview of Robotic Colorectal Surgery Adoption and Training in Brazil

**DOI:** 10.3390/medicina59091675

**Published:** 2023-09-17

**Authors:** Bruno Augusto Alves Martins, Oswaldo de Moraes Filho, Tiago Leal Ghezzi, Armando Geraldo Franchini Melani, Luis Gustavo Capochin Romagnolo, Hélio Moreira Júnior, João Pedro Pereira de Almeida, Sérgio Eduardo Alonso Araújo, João Batista de Sousa, Romulo Medeiros de Almeida

**Affiliations:** 1 Department of Colorectal Surgery, Hospital Universitário de Brasília, Brasilia 70840-901, Brazil; 2 Hospital Moinhos de Vento, Porto Alegre 90035-000, Brazil; 3 IRCAD America Latina, Surgical Staff Americas Medical Services, Rio de Janeiro 22775-001, Brazil; 4 Department of Colorectal Oncology Surgery, Barretos Cancer Hospital, IRCAD America Latina, São Paulo 14784-400, Brazil; 5 Department of Surgery, Colorectal Service, School of Medicine, Federal University of Goias, Goias 74605-050, Brazil; 6 School of Medicine, Centro Universitário do Planalto Central, Brasília 72445-020, Brazil; 7 Hospital Israelita Albert Einstein, São Paulo 05652-900, Brazil

**Keywords:** (MeSH terms): colorectal surgery, robotic surgical procedures, robotic training, minimally invasive surgical procedures

## Abstract

*Background and Objectives:* Robotic surgical systems have rapidly become integrated into colorectal surgery practice in recent years, particularly for rectal resections, where the advantages of robotic platforms over conventional laparoscopy are more pronounced. However, as with any technological advancement, the initial high costs can be a limiting factor, leading to unequal health service access, especially in middle- and lower-income countries. *Materials and Method:* A narrative review was conducted with the objective of providing an overview of the escalating adoption, current training programmes, and certification process of robotic colorectal surgery in Brazil. *Results:* Brazil has witnessed a rapid increase in robotic platforms in recent years. Currently, there are 106 robotic systems installed nationwide. However, approximately 60% of the medical facilities which adopted robotic platforms are in the Southeast region, which is both the most populous and economically prosperous in the country. The Brazilian Society of Coloproctology recently established clear rules for the training programme and certification of colorectal surgeons in robotic surgery. The key components of the training encompass theoretical content, virtual robotic simulation, observation, assistance, and supervised procedures in colorectal surgery. Although the training parameters are well established, no colorectal surgery residency programme in Brazil has yet integrated the teaching and training of robotic surgery into its curriculum. Thus far, the training process has been led by private institutions and the industry. *Conclusion*: Despite the fast spread of robotic platforms across Brazil, several challenges still need to be addressed to democratise training and promote the widespread use of these platforms. It is crucial to tackle these obstacles to achieve greater integration of robotic technology in colorectal surgery throughout the country.

## 1. Introduction

Robotic surgical systems were designed to overcome the limitations of laparoscopic surgery. Within a few years, the routine use of robotic surgery by varied surgical specialities has been remarkably fast. Some technical advantages of robotic surgery include stable and highly magnified three-dimensional vision, improved hand-eye coordination, a surgeon-controlled field, optimised ergonomics, EndoWrist instruments with seven degrees of freedom, motion scaling, and physiological tremor filtering [1,2].

Since the first robotic colectomy was performed in 2001 [3], robotic-assisted procedures have gained substantial popularity in colorectal surgery, particularly in rectal cancer resection. An analysis of the University HealthSystem Consortium Clinical Database, an administrative database comprising inpatient data from approximately 95% of non-profit medical centres in the United States, revealed that the proportion of robotic colorectal procedures increased from 2.6% in 2012 to 6.6% in 2015 [4].

Operating within narrow spaces, such as the pelvis, represents a significant challenge, and robotic platforms offer additional tools to effectively handle anatomical and technical adversities, enabling complex and precise dissections [2]. Moreover, the recent REAL trial has indicated that robotic surgery is associated with superior short-term outcomes for middle and low rectal cancer compared to conventional laparoscopic surgery. These benefits include reduced positive circumferential margin rates, fewer conversions to open surgery, and improved and faster postoperative recovery [5].

Despite these potential benefits, the high cost represents the main drawback of robotic surgery compared to conventional laparoscopic surgery [6]. Mean operative costs for robotic surgery in the treatment of rectal cancer are estimated to be nearly £800 higher than those associated with laparoscopic surgery [7].

As well recognised with other technological advancements, this barrier usually initiates health inequities as economic conditions may prevent technological access and availability, especially in middle- and low-income countries [8]. Additionally, a shortage of specialised surgeons, a lack of standardised training in surgical practice, and network issues may also contribute to the inequitable adoption of robotic surgery in these countries [9].

These socioeconomic constraints similarly impact the practice of colorectal surgery in middle- and low-income nations. There is a scarcity of training opportunities, a lack of dedicated units, and insufficient research funding. Moreover, a significant proportion of colorectal resections are still conducted using open surgical techniques, while the availability of laparoscopic and robotic systems remains far from universal [10].

Upon first glance, the adoption of robotics in middle- and low-income countries may appear utopian. However, when one contemplates the potential benefits, significant factors come to the forefront. The decline in surgical site infection rates could result in shortened hospital stays and reduced antibiotic consumption. A quicker postoperative recovery would enable an early return to daily activities, consequently boosting overall productivity. Moreover, robotic surgery permits physical separation between surgeons and medical staff from the patient, thereby diminishing the risk of transmitting infectious diseases [9].

Another potential advantage is linked to surgical training and mentoring. Telerobotic surgery enables experienced surgeons to demonstrate operative steps to less-experienced colleagues and trainees remotely, potentially offering real-time guidance to surgeons during operations. This method effectively overcomes geographical limitations, enabling robotic surgeries to be conducted in remote areas without the necessity of the proctor’s physical presence [9].

Brazil is the leading emerging country in Latin America and boasts the region’s highest population count. Despite being the leading economic market and a major driving force in developing new medical technologies in the region, Brazil still grapples with significant socioeconomic disparities and health inequities.

This narrative review aims to provide an overview of the escalating adoption, current training programmes, and certification process of robotic colorectal surgery in Brazil.

## 2. Adoption of Robotic Colorectal Surgery in Brazil

Surgical practice in Brazil is shaped within two distinct health system settings. The public sector, named Unified Health System (Sistema Único de Saúde-SUS), ensures free and universal access to healthcare for all Brazilian citizens. It is financed through general taxes and social contributions collected by the federal, state, and municipal governments. On the other hand, the private/supplementary sector encompasses a variety of services funded by private or public funds and/or health insurance companies [11,12].

Despite a public health system that theoretically would offer free access to the entire population, this system faces significant difficulties in terms of funding, resulting in deficient hospital infrastructure, limited training for healthcare workers, and slowness in incorporating new healthcare technologies. As a result, disparities in healthcare outcomes might be observed between different regions of the country, as the populations of economically undeveloped areas are expected to rely more commonly on the public health system, while people from more developed regions are more commonly assisted by paying for a private supplementary health plan [13].

These healthcare system discrepancies in Brazil are evident when we look at the distribution of robotic platforms in our hospitals. Private institutions have been by far at the forefront of implementing new healthcare technology.

**Figure 1:**
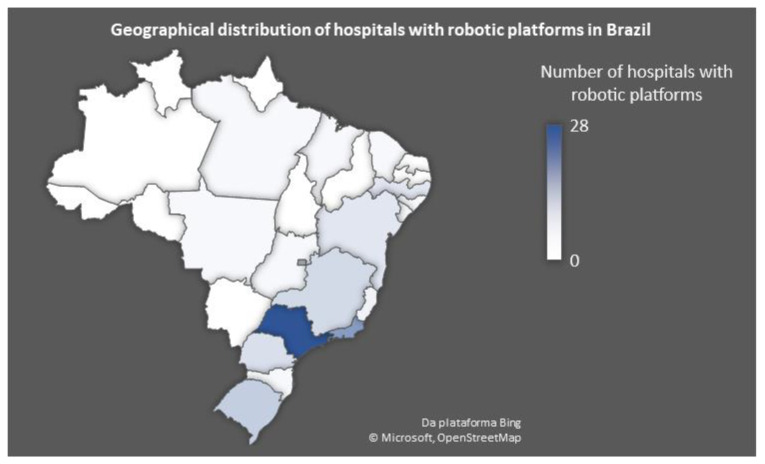
Geographical distribution of hospitals with robotic platforms in Brazil. São Paulo (dark blue) presents the highest number of robotic platforms, while only one hospital in the North region is equipped with robotic platforms.

Considering data about the world’s most widely used robotic platform today, (*da Vinci* ^®^ robotic system-Intuitive Surgical Inc., Sunnyvale, USA), there are currently 106 systems installed in Brazil. Among the 27 federative units, 17 have medical facilities that have adopted robotic platforms. Approximately 60% of these hospitals are in the Southeast region (see Figure 1), primarily in São Paulo, the most populous state in Brazil. In stark contrast, in the northern region, one of the least populated regions and with high poverty rates, only one hospital is equipped with a robotic platform [14].

**Figure 2:**
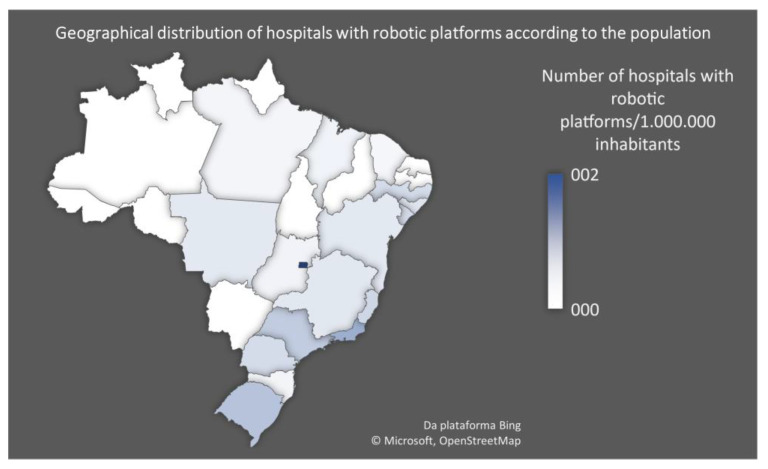
Geographical distribution of hospitals with robotic platforms in Brazil according to the population. Federal District (dark blue) presents the highest ratio of hospitals with robotic platforms per 1 million inhabitants.

Analysing the ratio between hospitals with a robotic platform and the population of the federative unit it belongs to, the Federal District, where the capital and seat of the federal government is located, boasts the highest ratio (see Figure 2). It presents more than two hospitals with robotic platforms for every 1 million inhabitants. Curiously, none of the hospitals with robotic platforms are public. Therefore, one can say that even in more developed areas, those who rely on the public health system are usually deprived of a high technological standard of care. Across the country, approximately 11.5% of medical facilities with a robotic platform are integrated into the public health system [14].

These disparities highlight the need to improve access to advanced medical technologies in the public healthcare system, especially in underprivileged regions, to ensure nationwide equitable healthcare services.

Even in the private sector, there are significant challenges to having access to robotic surgery, as these surgical procedures are not specifically discerned in the health events and covered procedures list established by the National Supplementary Health Agency [15]. Consequently, health insurance providers often question the liability to cover the cost of the procedure. Most hospitals end up charging the patient an extra fee (about $2500 US dollars) for using the robotic system and their specific instruments. These factors may hamper access to this advanced surgical technology as it remains financially challenging for some patients to avail themselves of the benefits of robotic-assisted surgery.

It has been estimated that, to date, around 118,000 robotic-assisted surgical procedures have been performed in Brazil [14]. Even though the exact percentage of colorectal procedures remains uncertain, there is undoubtedly a high demand of patients who may benefit from such robotic surgery in Brazil.

Colorectal cancer ranks third among the most frequent cancers in Brazil, with more than 45,000 new cases expected in 2023 [16]. Additionally, the incidence of inflammatory bowel disease rose from 9.4 cases per 100,000 in 2012 to 9.6 cases per 100,000 in 2020, and the prevalence increased from 30.0 to 100.1 cases per 100,000 [17]. It is also important to emphasise the high prevalence of diverticular disease and endometriosis, which account for more than 25% of surgical indications for minimally invasive colorectal operations in Brazil [18]. These figures indicate a significant health challenge and emphasise the importance of advanced surgical techniques, such as robotic-assisted procedures, to address these conditions effectively.

The very first reported case of colorectal resection surgery utilising a robotic-assisted surgical device in Brazil occurred in 2008. The procedure was to treat deep infiltrating endometriosis with rectal involvement in a 35-year-old female patient. The operation and postoperative recovery were uneventful [19]. Since then, there has been rapid adoption of robotic systems in colorectal surgery.

Retrospective studies conducted in Brazilian institutions have shown positive outcomes, reinforcing that robotic-assisted surgery is a reliable and effective option for managing colorectal pathological conditions, such as deep infiltrating bowel endometriosis [20] and rectal cancer [21].

Neme and collaborators (2013) conducted a retrospective analysis on ten patients who underwent robotic-assisted rectosigmoidectomy for deep infiltrating colorectal endometriosis. The study found that the patients had a mean length of stay of 3 days and a mean operative time of 157 min. There were no intraoperative or postoperative complications [20].

A group of researchers led by Denadai (2020) analysed the medical records of 102 people who had undergone robotic surgery for rectal cancer using the single-docking technique. The surgeries occurred between September 2014 and April 2018 at a single referral centre. The study found that 23.5% of patients experienced complications within 30 days of the procedure, and the mortality rate was 1.9%. Intraoperative complications occurred in 4.9% of patients, and only 1.9% of cases required conversion, and the average hospital stay was three days. The median number of lymph nodes harvested was fifteen [21].

Undoubtedly, the expansion of robotic colorectal surgery in Brazil has been remarkable. However, there is a pressing need to establish mechanisms that enhance the population’s access to this resource, particularly in the public healthcare scenario. We strongly believe that the expansion of robotic platform users in the market, associated with the upcoming new robotic platforms, will be a determining factor in reducing costs. It will ultimately make this technology more available to a more significant number of hospitals, especially for Public Institutions [1]. Ultimately, what we expect is to give the benefit of this technology to a broader segment of our population.

## 3. Robotic Colorectal Surgery Training and Qualification in Brazil

The rapid spread of robotic surgical platforms across major centres in Brazil has created a demand for training programmes for surgeons and other involved professionals in the process of robotic surgery, including clinical engineers, material centre staff, nurses, surgical scrub technicians, operating room coordinators, and circulating personnel.

Robotic surgery possesses distinctive features compared to laparoscopy, such as the absence of tactile feedback and the physical separation between the surgeon, the team, and the patient. As a result, comprehensive training and the attainment of an adequate learning curve are paramount to mastering this technology [22].

Proficiency in using instruments and equipment and a well-designed training programme prioritising patient safety and good outcomes are crucial in achieving positive clinical outcomes with new technology. Standardisation and a structured training programme can significantly shorten the often lengthy learning process that accompanies new technologies and techniques [23]. The core elements of most robotic-assisted colorectal surgery training programmes are theoretical knowledge, observation, simulation, and proctored training [24].

So far, the training process has been primarily led by private institutions that have acquired robotic surgical platforms and by Strattner^TM^ (Rio de Janeiro, Brazil), a partner of the Intuitive^TM^ company in Brazil. Currently, there are over 2500 surgeons trained to use the *da Vinci system* (Intuitive Surgical Inc., Sunnyvale, CA, USA) in Brazil [25]. This growing number of trained professionals reflects the increasing demand for expertise in utilising advanced robotic technology.

By recognising the need for a regulatory qualification in robotic surgery, the Brazilian Medical Association published a statement in December 2019 outlining the criteria for the robotic certification process in collaboration with surgical societies within their respective areas of expertise [26,27]. Subsequently, through Resolution number 2311/2022, the Federal Council of Medicine took steps to regulate robotic surgery in Brazil. It established the minimum prerequisites for a surgeon to perform a robotic procedure [28]. This vital statement aimed to ensure that surgeons involved in robotic-assisted surgeries own the qualifying needs and proper training, guaranteeing enhanced patient safety and an overall better and more uniform quality of robotic surgical practices in our country.

In agreement with the published rules established by the regulatory entities of medicine in Brazil, the Brazilian Society of Coloproctology recently issued regulations that order the certification process for robotic colorectal surgery. Among all the eligible criteria (see Table 1), completing at least ten fully robotic colorectal surgical procedures and accomplishing theoretical and practical training in robotic surgery stand out as key requirements [29].

The specifications of the theoretical-practical training established by the Brazilian Society of Coloproctology (see Table 2) include a minimum 15 h workload of theoretical content. Subsequently, the surgeon must complete at least 30 h of virtual reality simulator training. This is followed by training in the robotic system, known as the in-service session, which aims to assess the surgeon’s knowledge and competence in handling the robotic system. The next phase of the training involves observing and assisting in complex colorectal surgeries. Finally, the last step consists of performing at least ten complete robotic colorectal operations supervised by a proctor [29].

While the parameters for training are well established, the question arises: Who should take responsibility for providing training in robotic colorectal surgery? As mentioned earlier, up to now, the qualification process for surgeons in our country has been primarily led by the industry and private hospitals. To our knowledge, no medical residency program in coloproctology in Brazil has yet incorporated teaching and training robotic surgery into their curriculum. Similarly, no fellowship programmes in robotic colorectal surgery are established in public medical education networks.

The successful integration of robotics into colorectal surgery residency programs may face several hurdles. The primary challenge is the availability of a robotic system within the institution. Another hindrance is the absence of a mentor surgeon who is proficient in teaching robotic colorectal surgery. Furthermore, there is a need for a sufficient case volume to attain proficiency and ample time in the residency programme to learn a multitude of colorectal approaches. Additionally, the trainees’ participation in robotic cases may be limited by the lack of a dual console, which is essential in creating a secure learning environment [30]. This tool enables the proctor to gradually transfer control of the robotic instruments to the trainee through the functions “give and take” and “swap all”. The main drawback of the dual console is the additional cost, which is estimated at approximately $500,000 [22].

When juxtaposed with developed nations, a stark disparity becomes evident concerning integrating robotic surgery into colorectal residency training programmes. A survey administered to all colorectal surgery residency programmes in the United States and Canada in 2019, boasting a response rate of 64%, revealed that 98% of the responding institutions possessed a robotic surgical platform. Ninety-five percent of the programmes reported active participation of trainees in robotic procedures. Low anterior resection, abdominoperineal resection, and colectomy were reported to be the most frequently performed robotic procedures by trainees. Nearly 80% of the institutions reported the presence of a dual console, and approximately 75% of residency programmes had adopted robotics as an integral component of their training curriculum. The key facets of the training process included virtual robotic simulations, faculty-guided console time, and industry-sponsored training. Notably, most of these residency programmes were university-based or university-affiliated [30].

In Europe, fellowships in colorectal robotic surgery have been offered by the European Society of Coloproctology (ESCP) and Intuitive^TM^. During the fellowship, trainees are instructed to perform standard procedures in robotic colorectal surgery, such as rectopexy, colon resections, and low anterior resection [31].

Of note, The European Academy of Robotic Colorectal Surgery (EARCS) has formulated a structured training programme aimed at the safe adoption of robotic colorectal surgery. This programme encompasses simulation exercises, online modules, and hands-on and supervised training. An analysis of short-term clinical outcomes from 1130 robotic colorectal procedures across 26 European centres performed by trainees, graduates, and proctors has demonstrated that this standardised approach enables a feasible and secure learning process. Aside from operative time, blood loss, and length of stay, which were significantly lower in the proctor group, other outcomes such as reoperations, readmissions, mortality, anastomotic leaks, complications, and lymph node harvest were comparable between the three groups. Since 2014, surgeons from 15 European countries have graduated from EARCS [32].

In 2022, the Brazilian Society of Coloproctology sent out a national survey called “SBCP: Who Are We?” to all full and associate members of the society via email. The survey received responses from 680 colorectal surgeons, resulting in a response rate of 49%. The survey findings were presented at the Brazilian Congress of Coloproctology opening ceremony in the same year.

Out of all the participants, only 9.7% stated that they had received training in robotic surgery and integrated the technique into their practice. As many as 92.4% of the qualified surgeons were operating in major cities, indicating the concentration of robotic platforms in capital regions. Additionally, 16.8% of the respondents reported completing the training but had yet to incorporate the technique into their care practice. Regarding preferred surgical techniques, only 2.4% of respondents expressed a preference for robotic-assisted surgery over an open approach or laparoscopy in colonic resections and 3.9% in rectal resections.

These results indicate that, despite the fast spread of robotic platforms across Brazil, several challenges still need to be addressed to democratise training and promote the widespread use of these platforms. It is crucial to tackle these obstacles to achieve greater integration of robotic technology in colorectal surgery throughout the country.

## 4. Future Directions

Arguably, the most significant challenge facing Brazil concerning robotic surgery pertains to securing the financial means for adopting, implementing, and maintaining the latest technological advancements within the public healthcare system. Such an initiative would expand access to robotic surgery to a more extensive segment of the population, including those living in remote areas and impoverished conditions.

It is also anticipated that, as technology matures and new competitors enter the market, costs will decrease. This, in turn, will facilitate a more widespread deployment of robotic platforms and the subsequent democratisation of access.

Another strategy to enhance the affordability and accessibility of robotic surgery involves regionalising complex healthcare services by establishing specialised referral centres in specific areas such as rectal cancer, colon cancer, inflammatory bowel disease, polyposis, pelvic exenteration, pelvic floor disorders, and so forth. This healthcare configuration also generates a sufficient volume of robotic cases, thereby enhancing the expertise of both trainees and mentors. Ultimately, these referral centres may evolve into regional hubs for training, research, and specialised care.

The expansion of the number of robotic surgical platforms should be concomitant with an augmentation in the availability of training opportunities for surgeons and all personnel engaged in the execution of these surgical procedures.

It is imperative to incorporate the teaching of robotic surgery into the education and training of colorectal surgeons. Colorectal surgery residency programmes and fellowships play a pivotal role in this regard. Establishing a teaching network comprising mentor surgeons and training centres can significantly contribute to the safe and effective integration of robotic techniques. Ideally, the fundamentals of robotic surgery should be introduced during the general surgery residency, enabling trainees to grasp and practice the core aspects of this surgical technology.

The expanding utilisation of 5G communication technology opens opportunities for telesurgery and telementoring, allowing the proctor to supervise another surgeon or trainee in a remote location. In a country with vast geographical dimensions like Brazil, this tool can contribute to delivering high-quality healthcare to remote and isolated communities.

## 5. Conclusions

Incorporating robotic surgical platforms into surgical practice in Brazil has seen a significant rise in recent years. However, it is essential to note that there is a noticeable discrepancy in the distribution of this technology, with most of the robotic systems only being accessible to private institutions and larger economic centres. In the domain of colorectal surgery, there has been a rapid expansion, and to ensure quality standards, the Brazilian Society of Colorectal Surgery has recently established a set of guidelines for training and certification in robotic colorectal surgery. Nevertheless, the biggest challenges that need to be addressed are achieving a more balanced distribution of robotic platforms within the Brazilian healthcare system and developing comprehensive training programmes for surgical residents and coloproctology fellows.

## Figures and Tables

**Table 1:** Eligibility criteria for the qualification certificate in robotic colorectal surgery in Brazil according to the Brazilian Society of Coloproctology [29].

Eligibility Criteria for the Qualification Certificate in Robotic Colorectal Surgery in Brazil
1 The surgeon must hold a specialist title or Register of Medical Specialty Qualification in Coloproctology.
2 Membership in good standing with the Brazilian Society of Coloproctology is required.
3 Applicants must provide proof of theoretical-practical training in robotic surgery or hold console certification issued by Intuitive.
4 Proof of performing at least ten complete robotic colorectal surgeries.

**Table 2:** Minimum prerequisites for training programs in robotic colorectal surgery in Brazil, according to the Brazilian Society of Coloproctology [29].

Requirements for Training Programmes in Robotic Colorectal Surgery in Brazil
1 Fifteen hours of theoretical content.
2 Thirty hours of virtual reality simulator training.
3 A four-hour in-service session.
4 Observership phase in robotic colorectal procedures.
5 Participation as an assistant in robotic colorectal procedures.
6 Completion of at least ten robotic colorectal operations under the supervision of a proctor.

## Data Availability

No new data were created or analyzed in this study. Data sharing is not applicable to this article.
